# Reporting quality of surgical randomised controlled trials in head and neck cancer: a systematic review

**DOI:** 10.1007/s00405-021-06694-9

**Published:** 2021-02-19

**Authors:** Netanya Aarabi Canagarajah, George James Porter, Kurchi Mitra, Timothy Shun Man Chu

**Affiliations:** Faculty of Medical Sciences, Newcastle University, Framlington Place, Newcastle Upon Tyne, NE2 4HH UK

**Keywords:** Systematic review, Randomised controlled trials, Reporting quality, CONSORT, Head and neck cancer, Head and neck surgery

## Abstract

**Purpose:**

Randomised controlled trials (RCTs) are considered the gold standard for evaluating the efficacy of an intervention. However, previous research has shown that RCTs in several surgical specialities are poorly reported, making it difficult to ascertain if various biases have been appropriately minimised. This systematic review assesses the reporting quality of surgical head and neck cancer RCTs.

**Methods:**

A literature search of PubMed and Embase was performed. Papers were included if they reported RCTs which assessed a surgical technique used to treat or diagnose head and neck cancer published during or after 2011. The CONSORT 2010 checklist was used to evaluate the reporting quality of these trials.

**Results:**

41 papers were included. The mean CONSORT score was 16.5/25 (66% adherence) and the scores ranged from 7.5 (30%) to 25. The most common omissions were full trial protocol (found in 14.6%), participant recruitment method (22%) and effect size with a precision estimate for all outcome measures (29.3%). The full design and implementation of the randomisation methods were reported in 6 (14.6%). Papers published in journals which endorsed CONSORT had significantly higher scores (*p* = 0.02) and the journal impact factor was significantly correlated with CONSORT score (*p* = 0.01).

**Conclusion:**

We have identified several pieces of information that are underreported in surgical head and neck cancer RCTs. These omissions make understanding and comparing the methodologies and conclusions of RCTs more difficult. The endorsement of CONSORT by journals improved adherence, suggesting that wider adoption of the checklist may improve reporting.

**Supplementary Information:**

The online version contains supplementary material available at 10.1007/s00405-021-06694-9.

## Introduction

Randomised controlled trials (RCTs) are largely considered the gold standard in determining the efficacy of treatments and interventions in the medical field. The processes used in an RCT, such as randomisation and double-blinding, remove sources of bias which are inherent to other trial designs [1, 2]. According to the Oxford Centre for Evidence-based Medicine, RCTs are classified as level 1b in the Levels of Evidence [3].

Unless research is adequately reported, it can potentially waste billions in investment and detrimentally impact research and patient care [4]. While they may not reflect poor methodology, poor reporting can create doubt about the results and conclusions of both RCTs, and the systematic reviews of which they may be a part [5, 6]. Poor reporting is associated with bias in the estimation of intervention effectiveness, and limits the critical appraisal and results interpretation by the reader [4]. Poor reporting has the potential to affect healthcare decisions at all levels, from an individual patient to national public health policies [7].

In the 1990s, significant shortcomings were identified in the reporting quality of RCTs in general medicine and several specialties. For instance, a study conducted by Ah-See et al. [8] assessed 295 otolaryngology papers over a 30-year period (1966–1995) and identified an unsatisfactory overall mean score of 7.3 out of 12 (scoring proforma based on CONSORT and observations from existing ENT literature). Similar findings of historical poor reporting are documented in other disciplines as well [9, 10]. Efforts have been made to bridge this gap in the reporting quality of RCTs with the introduction of various guidelines such as The Consolidated Standards of Reporting Trials (CONSORT) statement [11].

The CONSORT statement was first conceptualised in 1993 after a group of medical journal editors, authors, epidemiologists and clinical trialists acknowledged the growing pile of evidence highlighting the poor reporting quality of RCTs. They developed the CONSORT Statement, comprising a numbered checklist that guides researchers on the various aspects of how RCTs are conducted and exposed flaws and inaccuracies in the reporting process [11]. The statement was published in 1996, revised in 2001 and 2010 to take into consideration new concerns.

However, the success of guidelines such as CONSORT depends entirely on the adherence to them. Agha et al. [9] looked at the compliance of 122 urological surgical RCTs in the period 2000–2003 (post-CONSORT) and obtained an average CONSORT score of 11.2 out of a possible 22. It is well documented that compliance to the CONSORT statement has been poor across several other disciplines as well [12, 13].

Surgical RCTs present certain unique practical and ethical difficulties, such as difficulties associated with blinding and the ethical concerns associated with “sham/placebo” studies [14]. This could contribute to poor reporting in the field, as illustrated by the median CONSORT(NPT) score of 27/42 in a study of randomised trials in surgery by Nagendran et al., which looked at 54 trials from 2011 [15]. The CONSORT Non-Pharmacological Treatments (CONSORT-NPT) extension was introduced in 2008 with the aim to remediate some of these issues. However, there is contention as to whether prospective RCTs and therefore, the CONSORT checklist, are appropriate methods of quality assurance for surgical trials [16]. Apart from the features mentioned above, surgical trials pose difficulties in patient recruitment, the uniqueness of every procedure and quality control, and the duration and cost needed to reach the primary end point [16].

To our knowledge, there were three published studies that focussed specifically on the reporting of surgical interventions in head and neck surgery. The first two studies restricted their findings to a few selected journals only and included papers from a limited time-frame (2011–2014) [17, 18]. The third selected papers from 1977 to 2012 and focussed mainly on the different reporting quality of papers written by surgeons versus non-surgeons [19]. There is, therefore, need for a wider and more current analysis of the literature base.

In summary, reporting all the relevant components of an RCT is important to elicit the full value of the study to clinicians. The primary objective of our study was to assess the compliance of the reporting of RCTs in head and neck cancer surgery to the 2010 CONSORT checklist and highlight specific shortcomings in reporting quality. Additionally, we analysed the relation between the 5-Year Impact Factor and CONSORT score, and compared the adherence in journals that did and did not endorse CONSORT.

## Methods

This systematic review was carried out in accordance with the Preferred Reporting Items for Systematic Reviews and Meta-Analyses (PRISMA) guidelines [20].

### Literature search

A structured literature search was conducted using PubMed and Embase via OvidOnline by two authors (NAC and GJP). The search was performed on 18/02/2020. Both databases listed studies up to week six of 2020, from January 1954 for PubMed and January 1988 for Embase.

A full literature search strategy can be found in ESM Appendix A1. In brief, terms such as “Head and Neck Cancer”, “Head and Neck Surgery” and “Surgery” were exploded and combined with the Boolean operator ‘AND’. The results were then limited to RCTs either via limits native to the search engine or via including “Randomized Controlled Trial” in the search and combining with the Boolean operator ‘AND’. Results were then limited to after 2011, as papers published prior to this date were likely to be written before the updated CONSORT statement was published in 2010.

### Paper selection

All articles from the initial search underwent title and abstract screening by GJP, TC and KM. Prospective RCTs where a surgical technique used to treat or diagnose head and neck cancer was the intervention in at least one of the arms were included. Studies were excluded if they were on cadavers or animals, were not reported in English or pertained to non-surgical, peri-operative, cosmetic or ophthalmic interventions not relating to head and neck cancer.

### Data extraction

A data extraction proforma, based on the CONSORT 2010 guideline, was made by TC. Each checklist item and sub-item was scored with a simple yes/no answer. Total scores of items 1–25 were then calculated, with a point for each item being granted if all sub-sections were scored as yes or half a point if one out of the two sub-sections were reported. Authors noted down the reasons for deducted points for further analysis. Raters were not blinded.

### Rater training

The first three papers were read sequentially by both NAC and GJP. Any discrepancies in scoring between the two authors were discussed until a consensus was reached. If agreement could not be reached, a third author (TC) was consulted for the final decision. After this, NAC and GJP produced an agreed guideline to scoring, which they used to score the next paper and once again compared results.

After consensus was reached, the authors (NAC and GJP) each read and scored half of the papers. A further ten papers were scored by both raters to check overall rater agreement after the moderation processes.

### Statistical analysis

Statistical analysis was carried out using SPSS version 26 (IBM, Armonk, NY, USA) and *p* < 0.05 was considered statistically significant. Percentage agreement between the two raters was used to identify the impact of the moderation processes as well as identify any major disagreements. Journal impact factor was harvested from Clarivate Analytics [21]. The correlation between journal impact factor and mean adherence to CONSORT was assessed via Spearman’s rank correlation coefficient. An independent samples *T* test was used to compare the differences in CONSORT scores between journals which did and did not endorse CONSORT, as the data met all the relevant assumptions including normality and homogeneity of variance. The same test was used to assess the difference in impact factors between journals which did and did not endorse CONSORT. Where appropriate, 95% confidence intervals (CI) were also calculated.

## Results

### Papers included

**Fig. 1:**
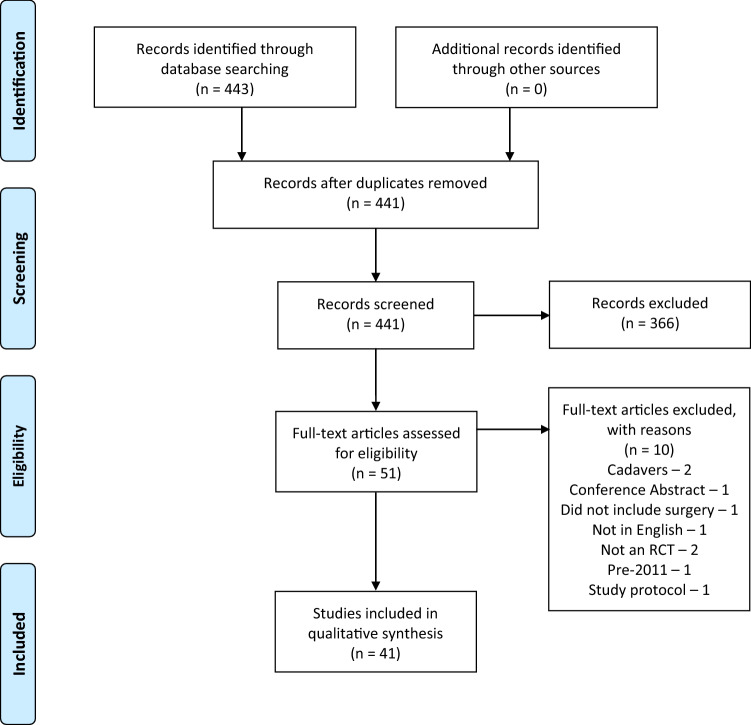
A flow chart adapted from the PRISMA guidelines illustrating the paper screening process and the reasons for exclusion during full-text screening [20]

The structured literature search identified 443 articles. After deduplication and title and abstract screening, 51 articles were subjected to full-text assessment, of which 10 were excluded. Forty-one papers that fulfilled the inclusion criteria were included in our analysis (ESM Appendix A2). Figure 1 outlines paper selection via the PRISMA flow diagram.

### Agreement among parallel readers

During the first round of scoring by NAC and GJP, the raw percentage agreement was 65.8%. After producing an agreed guideline, the raw percentage agreement increased to 73.0%. After further discussion and revision of the guideline, the agreement between the two raters was 92.01% in the ten papers scored after the moderation processes.

### Sample characteristics

The 41 included papers were from 26 journals. There were three journals contributing more than 2 papers, these were European Archives of Oto-Rhino-Laryngology (6 papers), Head and Neck—Journal for the Sciences and Specialities of the Head and Neck (4 papers) and Otolaryngology—Head and Neck Surgery (3 papers).

### Overall adherence to CONSORT checklist

**Fig. 2:**
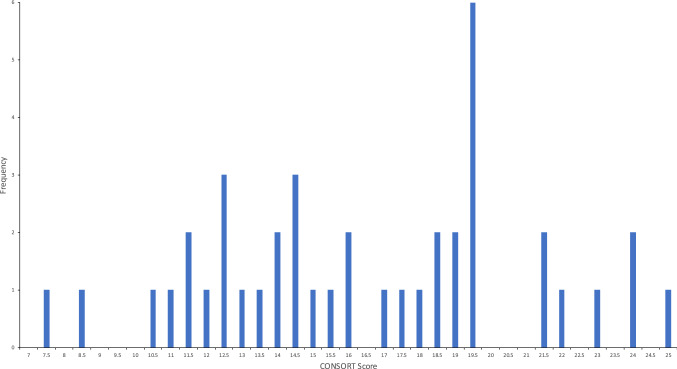
A bar chart illustrating the frequencies of the total CONSORT scores from all 41 RCTs

Only 1 paper reported all 25 items (100% adherence) of the CONSORT checklist. The scores ranged from 7.5 (30% adherence) to 25 (100%). The 41 papers had a mean score of 16.5 [66% adherence; 95% CI (60.7–71.3%)] and a median score of 16 (64%). Figure 2 shows a bar chart with frequencies of total scores from all papers.

### Adherence to individual items of checklist

The CONSORT checklist items of scientific background, scientific objectives and interpretation of results were present in all 41 papers (100%). Items which were poorly reported include identifying personnel responsible for the execution of different aspects of the trial (9/41, 22%) and the estimated effect size and its precision of results (12/41, 29.3%). Table 1 outlines the reporting frequency of each checklist item.

**Table 1:** The frequency and adherence of RCTs to individual items of CONSORT 2010 checklist, in order of increasing fulfillment

Item	Description	Frequency	Adherence (%)
24	Where the full trial protocol can be accessed, if available	6/41	14.6
4a	Eligibility criteria for participants	8/41	19.5
10	Who generated the random allocation sequence, who enrolled participants, and who assigned participants to interventions	9/41	22.0
17a	For each primary and secondary outcome, results for each group, and the estimated effect size and its precision (such as 95% confidence interval)	12/41	29.3
9	Mechanism used to implement the random allocation sequence (such as sequentially numbered containers), describing any steps taken to conceal the sequence until interventions were assigned	13/41	31.7
3a	Description of trial design (such as parallel, factorial) including allocation ratio	16/41	39.0
8b	Type of randomisation; details of any restriction (such as blocking and block size)	16/41	39.0
25	Sources of funding and other support (such as supply of drugs), role of funders	17/41	41.5
23	Registration number and name of trial registry	19/41	46.3
14b	Why the trial ended or was stopped	21/41	51.2
4b	Settings and locations where the data were collected	22/41	53.7
7a	How sample size was determined	23/41	56.1
8a	Method used to generate the random allocation sequence	24/41	58.5
21	Generalisability (external validity, applicability) of the trial findings	24/41	58.5
14a	Dates defining the periods of recruitment and follow-up	26/41	63.4
19	All important harms or unintended effects in each group	26/41	63.4
16	For each group, number of participants (denominator) included in each analysis and whether the analysis was by original assigned groups	27/41	65.9
1a	Identification as a randomised trial in the title	28/41	68.3
13b	For each group, losses and exclusions after randomisation, together with reasons	28/41	68.3
20	Trial limitations, addressing sources of potential bias, imprecision, and if relevant, multiplicity of analyses	28/41	68.3
17b	For binary outcomes, presentation of both absolute and relative effect sizes is recommended	29/41	70.7
11a	If done, who was blinded after assignment to interventions (for example, participants, care providers, those assessing outcomes) and how	32/41	78.0
13a	For each group, the numbers of participants who were randomly assigned, received intended treatment, and were analysed for the primary outcome	33/41	80.5
6a	Completely defined pre-specified primary and secondary outcome measures, including how and when they were assessed	34/41	82.9
15	A table showing baseline demographic and clinical characteristics for each group	34/41	82.9
12b	Methods for additional analyses, such as subgroup analyses and adjusted analyses	38/41	92.7
18	Results of any other analyses performed, including subgroup analyses and adjusted analyses, distinguishing pre-specified from exploratory	38/41	92.7
3b	Important changes to methods after trial commencement (such as eligibility criteria), with reasons	39/41	95.1
11b	If relevant, description of the similarity of interventions	39/41	95.1
12a	Statistical methods used to compare groups for primary and secondary outcomes	39/41	95.1
1b	Structured summary of trial design, methods, results, and conclusions	40/41	97.6
5	The interventions for each group with sufficient details to allow replication, including how and when they were actually administered	40/41	97.6
6b	Any changes to trial outcomes after the trial commenced, with reasons	40/41	97.6
2a	Scientific background and explanation of rationale	41/41	100
2b	Specific objectives or hypotheses	41/41	100
7b	When applicable, explanation of any interim analyses and stopping guidelines	41/41	100
22	Interpretation consistent with results, balancing benefits and harms, and considering other relevant evidence	41/41	100

### CONSORT Endorsement and CONSORT Score

**Fig. 3:**
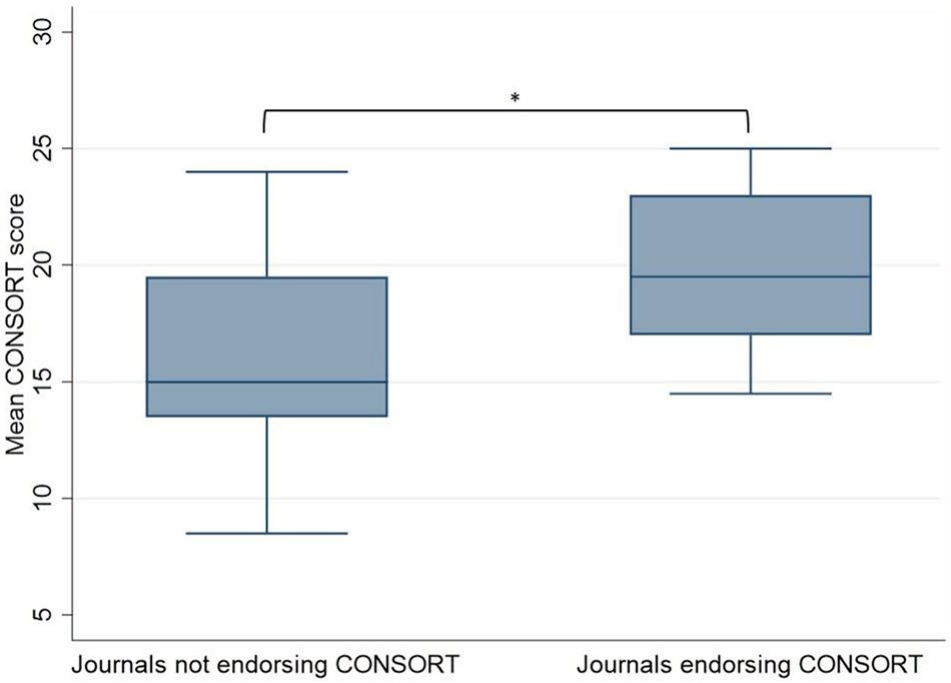
A box plot to compare adherence to the CONSORT checklist (%) between journals that endorse the use of CONSORT and those that do not. * Indicates statistical significance at *P* < 0.05

Only 9 out of the 26 journals officially endorse the use of CONSORT [22]. An Independent samples *T*-test was carried out to compare scores from journals which endorsed the use of CONSORT and those that did not. This resulted in a significant difference with *t* = 2.45 (95% CI 2.48–29.13), *p* = 0.02*.* Figure 3 shows a box plot comparing CONSORT adherence between these two variables.

### Journal impact factor

The Clarivate Journal Citation Reports provided us with the 2018 5-year impact factor for these journals [21]. At the time of writing, the Journal Citation Reports 2019 data update had been released in late June 2020 but was still subject to modifications. Therefore, we continued to use the 2018 data [23].

Three journals making up four papers did not have a 5-year impact factor. These were the International Journal of Surgical Oncology (1 paper), Journal of Laparoendoscopic and Advanced Surgical Techniques (2 papers) and Saudi Journal of Anaesthesia (1 paper).

**Fig. 4:**
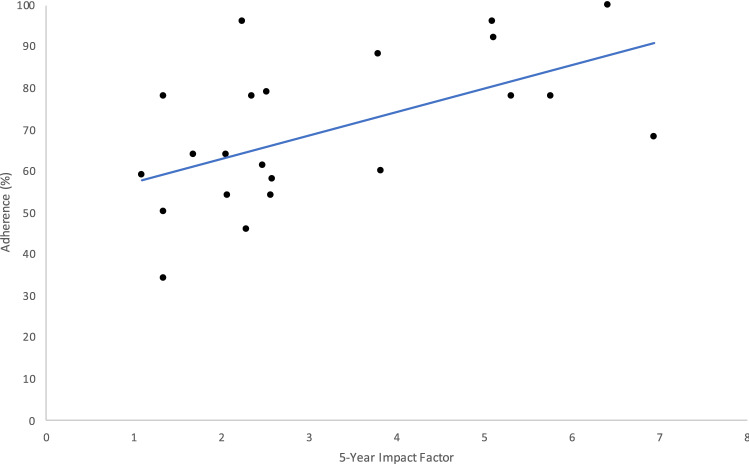
A scatter plot to show the 5-year impact factor vs adherence (%) to CONSORT checklist

There was a statistically significant positive correlation between the journals’ 5-year impact factor and CONSORT score with Spearman *ρ* = 0.52 and *p* = 0.01. This is illustrated by the scatter plot in Fig. 4. All journals had a 5-year impact factor less than 7 except New England Journal of Medicine (NEJM), which had a 5-year impact factor of 70.3 and was an outlier that was excluded from the graph.

The mean impact factor of journals which endorsed CONSORT was 11.4 (6.71 when removing the New England Journal of Medicine, NEJM, as an outlier) while the mean impact factor for journals which did not endorse CONSORT was 2.22. A *t*-test revealed that this was not a statistically significant difference (*p* = 0.084) even when NEJM was included (*p* = 0.071).

## Discussion

With an overall average CONSORT score of 16.5 (66% adherence) from 41 papers, it is clear that there is room for improvement in the reporting quality of surgical head and neck cancer RCTs. Only one paper reported all 25 items (100% adherence) and the lowest-scoring paper had 30% adherence.

This study has identified certain pieces of information which are routinely under-reported in manuscripts pertaining to RCTs in the field of head and neck cancer surgery, which limits the application of their results in clinical practice. In particular, the design and implementation of randomisation was only reported in 3 (7.3%) of the included studies. This is problematic as information on randomisation is a crucial requirement for reporting randomised controlled trials. This omission prevents readers from fully judging and understanding the reliability of findings. Furthermore, the eligibility criteria and recruitment method for participants was only explained completely in 8 papers (19.5%). Finally, precision estimates for the effect sizes of interventions were only presented in 12 (29.3%) of the included papers.

Moreover, several pieces of information designed to reduce ‘cherry picking’ of results are under-reported. These include full trial protocols (reported in 14.6%), reason for trial cessation (51.2%), trial registration (46.3%) and whether analysis was by intention-to-treat (65.9%). Inclusion of these data helps ensure that all information harvested, and analyses conducted by the trial are reported. Without this information, it is possible for authors to exclude participants or run analyses in such a way to magnify the observed differences between the trial arms. The reason RCTs are seen as a ‘gold standard’ of investigation is their ability to exclude biases, and the reporting of this information is important in this regard. Similarly, the sources of funding and roles of sponsors in a trial was only reported in 41.5% of the trials. This is a potential source of bias which should be explicitly stated in the text.

Several items were very well reported. Scientific background and rationale, objectives, consistent and interpretation of results were found in all studies. The use of the CONSORT flow diagram was very prevalent throughout the included studies as well as clear descriptions of the interventions used. However, these items are integral to readers’ understanding of the trial and therefore their inclusion is to be expected.

Articles from journals which endorsed the CONSORT guidelines had greater mean CONSORT adherence. This is to be expected but also implies that the reporting of RCTs would improve if more journals required CONSORT adherence. This supports the wider adoption of the CONSORT checklist in medical journals. Articles from high impact factor journals also had better CONSORT adherence. However, journals which endorsed CONSORT had a higher mean impact factor and while this difference was not significant (*p* = 0.07), this likely confounded the relationship.

### Comparison with existing literature

The main omissions identified by this study are not unique to head and neck cancer surgical RCTs. Agha et al. [9] conducted a systematic review of surgical RCTs with a special focus on urological trials in 2007. From their 90 included urological studies, the most frequent omissions were on the topic of randomisation, with no studies reporting how their randomisation sequence was implemented. They also found that only 20% of trials reported their sources of funding [9]. This is concordant with two reviews of reporting quality in plastic surgery RCTs, which both found that information on randomisation was poorly reported, with one study reporting compliance of 11% while the other found the information in just 2.4% of included studies [24, 25]. Equally, a review of 120 surgical RCTs found that none included information on randomisation implementation [26].

Items such as trial registration and full trial protocol have been shown to be poorly reported across a range of surgical specialties [9, 26]. Several trials included in our study did not report precision estimates for all effect sizes. This was replicated by a systematic review of orthopaedic RCT reporting quality [27]. In summary, much of the information that was routinely omitted in surgical head and neck cancer RCTs has been shown to be routinely omitted in other surgical specialities.

Head and neck cancer surgery was chosen because it is a rapidly evolving field and the evaluation of new techniques forms a cornerstone of evidence-base medicine. Despite this, there are several studies in similar areas which serve as useful comparisons. Huang et al. [17] conducted a systematic review of medical and surgical otolaryngology RCTs from a set of specific journals between 2010 and 2014. They found that only 6.5% of included papers described randomisation fully and 32.4% reported the effect size and precision. Carlton et al. [19] also reviewed the reporting quality of surgical head and neck cancer RCTs. However, their results were from 1977 to 2012, a considerably older sample than ours. They also found that randomisation was poorly reported, with the individual who implemented the randomisation sequence being reported in only 2.6% of their included papers [19]. In summary, several similar reviews to ours have found comparable results, implying that these omissions are consistently present across the literature base.

Journals which endorsed the use of CONSORT had a higher mean CONSORT adherence. This has been replicated in several studies [28], something which adds weight to the argument that endorsement of CONSORT is a viable method on the part of journals to increase reporting quality of trials.

It should be noted that direct comparisons of CONSORT scores between different studies can be unhelpful as different authors have interpreted CONSORT in different manners. Indeed, Hays et al. [29] stated directly that the CONSORT statement is open to significant interpretation and there is a need to reduce the CONSORT checklist ambiguity to improve adherence. This is also evident in the study by Huang et al. [17] who reported inter-rater reliability between their authors as 0.32 using Cohen’s *κ* and observed agreement of 0.87. CONSORT was not designed as a checklist to supply a quality score, so raw scores should be interpreted with caution [7].

Some have also stated that the CONSORT checklist is an inappropriate way of judging the reporting quality of surgical trials, and newer methods may be required to ensure reporting quality [16]. Although this could partly justify the low CONSORT scores seen throughout published literature on the topic, reporting quality appears low with major omissions such as randomisation and funding seen in many studies. Therefore, while valid criticisms can be made in the use of CONSORT to judge reporting quality, these do not invalidate the claim that more work is needed in this area to ensure adequate RCT reporting. In our study, we have used CONSORT to highlight routinely under-reported pieces of information in this literature base.

### Limitations of methodology

CONSORT also has an extension for RCTs concerning non-pharmacological treatments (NPTs). We did not use this because many studies included in our review involved both surgical and non-surgical interventions. As the latest CONSORT-NPTs version was published in 2017, limiting results to after this date would considerably limit our sample size. Also, the previous version of the CONSORT-NPTs did not include several important items, such as the source of funding.

Our literature search was conducted in English and only English language results were included. Also, while we collected data on whether a journal endorsed CONSORT, we did not record when it endorsed CONSORT in relation to the publication of the included studies. Therefore, it is unclear if the journal endorsed CONSORT at the time of publication. Given that the first iteration of CONSORT was published in 1996, this is unlikely to have made a large impact but is a potential inaccuracy.

Interrater agreement was high in our study and we had several measures to reduce individual biases, such as co-scoring of papers both before and after the main body was analysed. Despite this, we cannot completely exclude bias between the two raters.

## Conclusion

We have identified several pieces of information that are routinely underreported in surgical head and neck cancer RCTs. These make the comparison of methodologies of RCTs and the clinical application of trial results difficult due to missing information. In particular, detail on the randomisation processes used as well as the funding sources are key requirements for ensuring that a trial has minimised biases, and the omission of these is especially problematic.

Endorsement of CONSORT by journals improved adherence, suggesting that wider adoption of the checklist may improve reporting. There are several papers assessing the reporting quality of RCTs in various surgical specialties. Many of the findings of such papers are similar with detail on randomisation and full reporting of precision measures for effect sizes being common omissions. Given these reciprocal findings across manuscripts over several years it is possible that more explicit guidance from journals, academic bodies and CONSORT is required to improve reporting in these areas. For example, clinical academic departments, as well as journal editorial offices, could release explicit guidance on how to appropriately design, implement and report randomisation methods as well as the importance of reporting protocols and the roles of funders in the research.

## Supplementary Information

Below is the link to the electronic supplementary material.

Appendix A1 Full literature search strategy for the systematic review (PDF 39 KB)

Appendix A2 Details of the included studies (PDF 65 KB)

## Data Availability

Not applicable.
