# Supplementary material for: Reporting quality of surgical randomised controlled trials in head and neck cancer: a systematic review
Source: Eur Arch Otorhinolaryngol. 2021 Feb 19;278(11):4125–33. doi: 10.1007/s00405-021-06694-9 (PMC8486722; doi:10.1007/s00405-021-06694-9)
Supplement: Supplementary file 1 — Appendix A1 Full literature search strategy for the systematic review (PDF 39 KB) [file 405_2021_6694_MOESM1_ESM.pdf]

Reporting Quality of Surgical Randomised Controlled Trials in Head and Neck Cancer: A Systematic Review

European Archives of Oto-Rhino-Laryngology

Netanya Aarabi Canagarajah*[1], George James Porter*[1], MRes, Kurchi Mitra[1], Timothy Shun Man Chu[1], MRes.
[1]Faculty of Medical Sciences, Newcastle University, Framlington Place, Newcastle Upon Tyne, NE2 4HH, United Kingdom.

Contact: T.Chu1@newcastle.ac.uk

Full literature search strategy for the systematic review
Ovid: Embase 1988 to 2020 Week 07

1.

1: (Head and Neck Cancer).mp. [mp=title, abstract, heading word, drug trade name, original title, device manufacturer, drug manufacturer, device trade name, keyword, floating subheading word, candidate term word]	53771
exp "head and neck surgery"/	125209
1 and 2	4412
limit 3 to randomized controlled trial	102

2.

(Head and Neck Cancer).mp. [mp=title, abstract, heading word, drug trade name, original title, device manufacturer, drug manufacturer, device trade name, keyword, floating subheading word, candidate term word]	53771
(Head and Neck Surgery).mp. [mp=title, abstract, heading word, drug trade name, original title, device manufacturer, drug manufacturer, device trade name, keyword, floating subheading word, candidate term word]	17249
Randomised Controlled Trial.mp. or randomized controlled trial/	583212
1 and 2 and 3	53

3.

(Head and neck cancer).mp. [mp=title, abstract, heading word, drug trade name, original title, device manufacturer, drug manufacturer, device trade name, keyword, floating subheading word, candidate term word]	53771
(Head and neck surgery).mp. [mp=title,	17249

abstract, heading word, drug trade name, original title, device manufacturer, drug manufacturer, device trade name, keyword, floating subheading word, candidate term word]	
1 and 2	2017
Limit 3 to randomized controlled trial	52

4.

(Head and Neck Cancer).mp. [mp=title, abstract, heading word, drug trade name, original title, device manufacturer, drug manufacturer, device trade name, keyword, floating subheading word, candidate term word]	53713
Limit 1 to randomized controlled trial	1682
Exp “Head and Neck Surgery”/	125101
1 and 2 and 3	101

5.

1: (Head and Neck Cancer).mp. [mp=title, abstract, heading word, drug trade name, original title, device manufacturer, drug manufacturer, device trade name, keyword, floating subheading word, candidate term word]	53713
2: Limit 1 to randomized controlled trial	1682
3: “head and neck surgery”/	10205
4: limit 3 to randomized controlled trial	143
5: 1 and 2 and 3 and 4	41

6.

1: exp randomized controlled trial/	575875
2: (head and neck surgery).mp. [mp=title, abstract, heading word, drug trade name, original title, device manufacturer, drug manufacturer, device trade name, keyword, floating subheading word, candidate term work]	17233
3: (head and neck cancer).mp. [mp=title, abstract, heading word, drug trade name, original title, device manufacturer, drug manufacturer, device trade name, keyword, floating subheading word, candidate term word]	53713
4: 1 and 2 and 3	52

PubMed

(((head and neck surgery)) AND (head and neck cancer)) AND randomized controlled trial

Clinical Trial limit applied.
(((head and neck cancer)) AND (head and neck surgery)) AND RCT
Clinical Trial limit applied.
(((head and neck cancer)) AND surgery) AND RCT
Clinical Trial limit applied.
Limited to papers after 1990.
