# Supplementary material for: Reporting quality of surgical randomised controlled trials in head and neck cancer: a systematic review
Source: Eur Arch Otorhinolaryngol. 2021 Feb 19;278(11):4125–33. doi: 10.1007/s00405-021-06694-9 (PMC8486722; doi:10.1007/s00405-021-06694-9)
Supplement: Supplementary file 2 — Appendix A2 Details of the included studies (PDF 65 KB) [file 405_2021_6694_MOESM2_ESM.pdf]

Reporting Quality of Surgical Randomised Controlled Trials in Head and Neck Cancer: A Systematic Review

European Archives of Oto-Rhino-Laryngology

Netanya Aarabi Canagarajah*[1], George James Porter*[1], MRes, Kurchi Mitra[1], Timothy Shun Man Chu[1], MRes.
[1]Faculty of Medical Sciences, Newcastle University, Framlington Place, Newcastle Upon Tyne, NE2 4HH, United Kingdom.

Contact: T.Chu1@newcastle.ac.uk

	**Title**	**First Author**	**Year**	
1.	The utility of the harmonic scalpel in selective neck dissection: A prospective, randomized trial	Walen SG	2011	Otolaryngology - Head and Neck Surgery
2.	Total thyroidectomy with ultrasonic scalpel: a multicenter, randomized controlled trial	Kowalski LP	2012	Head & Neck
3.	A double blind randomised trial of IIb or not IIb neck dissections on electromyography, clinical examination, and questionnaire-based outcomes: A feasibility study	Parikh S	2012	British Journal of Oral and Maxillofacial Surgery
4.	Comparing use of the Sonopet((R)) ultrasonic bone aspirator to traditional instrumentation during the endoscopic transsphenoidal approach in pituitary tumor resection	Baddour HM	2013	International Forum of Allergy & Rhinology

5.	Carbon dioxide laser fiber for the excision of oral leukoplakia	Chee M	2013	Annals of Otology, Rhinology & Laryngology
6.	Harmonic scalpel versus conventional haemostasis in neck dissection: a prospective randomized study	Ferri E	2013	International Journal of Surgical Oncology
7.	The efficacy of the Harmonic scalpel in neck dissection: A prospective randomized study	Shin YS	2013	The Laryngoscope
8.	Voice quality after treatment of early vocal cord cancer: a randomized trial comparing laser surgery with radiation therapy	Aaltonen LM	2014	International Journal of Radiation Oncology, Biology, Physics
9.	Use of prototyping in preoperative planning for patients with head and neck tumors	Pires de Farias T	2014	Head & Neck
10.	Endoscopic Resection for Benign Parotid Tumor Through a Cosmetic Retroauricular Incision with Water Sac Establishing Operative Space: A New Approach	Yan Y	2015	Journal of Laparoendoscopic & Advanced Surgical Techniques – Part A
11.	Long-term results of a randomized phase III trial of TPF induction chemotherapy followed by surgery and radiation in locally advanced oral	Zhong LP	2015	Oncotarget

	squamous cell carcinoma			
12.	Utility of recorded guided imagery and relaxing music in reducing patient pain and anxiety, and surgeon anxiety, during cutaneous surgical procedures: A single-blinded randomized controlled trial	Alam M	2015	Journal of the American Academy of Dermatology
13.	Improving the rate of negative margins after surgery for oral cavity squamous cell carcinoma: A prospective randomized controlled study	Amit M	2016	Head & Neck
14.	Drainless Parotidectomies versus Conventional Parotidectomies: Randomised Control Study on Efficacy and Safety	Chua DY	2016	Annals Academy of Medicine Singapore
15.	Effectiveness of an (18)F-FDG-PET based strategy to optimize the diagnostic trajectory of suspected recurrent laryngeal carcinoma after radiotherapy: The RELAPS multicenter randomized trial	deBree R	2016	Radiotherapy and Oncology
16.	Harmonic scalpel impact on blood loss and operating time in major head and neck surgery: a randomized clinical	Fritz DK	2016	Journal of Otolaryngology - Head and Neck Surgery

	trial			
17.	Intraoperative goal-directed hemodynamic management in free tissue transfer for head and neck cancer	Hand WR	2016	Head & Neck
18.	The impact of tissue glue in wound healing of head and neck patients undergoing neck dissection	Huang CW	2016	European Archives of Oto-Rhino-Laryngology
19.	PET-CT surveillance versus neck dissection in advanced head and neck cancer	Mehanna H	2016	New England Journal of Medicine
20.	Prospective analysis of functional swallowing outcome after resection of T2 glottic carcinoma using transoral laser surgery and external vertical hemilaryngectomy	Nasef HO	2016	European Archives of Oto-Rhino-Laryngology
21.	Methylene blue staining in the parotid surgery: Randomized trial, 144 patients	Vaiman M	2016	American Journal of Otolaryngology
22.	Effects of free fat grafting on the prevention of Frey's syndrome and facial depression after parotidectomy: A prospective randomized trial	Wang S	2016	The Laryngoscope
23.	Potential role for carbon nanoparticles to guide central neck	Yu W	2016	Surgery

	dissection in patients with papillary thyroid cancer			
24.	Potential role of carbon nanoparticles in protection of parathyroid glands in patients with papillary thyroid cancer	Yu W	2016	Medicine (Baltimore)
25.	The effectiveness of myringotomy and ventilation tube insertion versus observation in post-radiation otitis media with effusion	Charusripan P	2017	European Archives of Oto-Rhino-Laryngology
26.	Transaxillary gasless endoscopic thyroidectomy versus conventional open thyroidectomy: a randomized study	Jantharapattana K	2017	European Archives of Oto-Rhino-Laryngology
27.	A prospective randomised trial of LigaSure Small Jaw versus conventional neck dissection in head and neck cancer patients	Lin WJ	2017	Clinical Otolaryngology
28.	PET-NECK: A multicentre randomised Phase III non-inferiority trial comparing a positron emission tomography-computerised tomography-guided watch-and-wait policy with planned neck dissection in the management of locally advanced (N2/N3) nodal	Mehanna H	2017	Health Technology Assessment

	metastases in patients with squamous cell head and neck cancer			
29.	Comparison of Output Volume Thresholds for Drain Removal After Selective Lateral Neck Dissection: A Randomized Clinical Trial	Tamplen ML	2017	JAMA Otolaryngology – Head & Neck Surgery
30.	Neck dissection with harmonic scalpel and electrocautery? A randomised study	Verma RK	2017	Auris Nasus Larynx
31.	The clinical diagnostic value of target biopsy using narrow-band imaging endoscopy and accurate laryngeal carcinoma pathologic specimen acquisition	Yang Y	2017	Clinical Otolaryngology
32.	Comparison Between Video-Assisted and Open Lateral Neck Dissection for Papillary Thyroid Carcinoma with Lateral Neck Lymph Node Metastasis: A Prospective Randomized Study	Zhang D	2017	Journal of Laparoendoscopic & Advanced Surgical Techniques – Part A
33.	Submental liposuction for the management of lymphedema following head and neck cancer treatment: A randomized controlled trial	Alamoudi U	2018	Journal of Otolaryngology - Head and Neck Surgery

34.	Combined use of a nanocarbon suspension and (99m)Tc-MIBI for the intra-operative localization of the parathyroid glands	Chen J	2018	American Journal of Otolaryngology
35.	Facial nerve dysfunction after superficial parotidectomy with or without continuous intraoperative electromyographic neuromonitoring: a prospective randomized pilot study	Graciano AJ	2018	European Archives of Oto-Rhino-Laryngology
36.	A randomized controlled trial comparing C Mac D Blade and Macintosh laryngoscope for nasotracheal intubation in patients undergoing surgeries for head and neck cancer	Hazarika H	2018	Saudi Journal of Anaesthesia
37.	Effect of goal-directed haemodynamic therapy in free flap reconstruction for head and neck cancer	Kim HJ	2018	Acta Anaesthesiologica Scandinavica
38.	2b or not 2b? Shoulder function after level 2b neck dissection: A double-blind randomized controlled clinical trial	Dziegielewski PT	2019	Cancer
39.	Donor-site morbidity after harvesting of	Halama D	2019	Journal of Cranio-Maxillofacial

	radial forearm free flaps-comparison of vacuum-assisted closure with conventional wound care: A randomized controlled trial			Surgery
40.	Effects of an antiadhesive agent on functional recovery of the greater auricular nerve after parotidectomy: a double-blind randomized controlled trial	Nam IC	2019	European Archives of Oto-Rhino-Laryngology
41.	Endoscopic dilatation improves long-term dysphagia following head and neck cancer therapies: a randomized control trial	Wu PI	2019	Diseases of the Esophagus
